# Mixed-method assessment of caregiver feeding practices in early care and education centres during COVID-19

**DOI:** 10.1017/S1368980022002452

**Published:** 2022-11-08

**Authors:** Joanna Farrer Mackie, Heewon Gray, David Himmelgreen, Jennifer Marshall, Abbey Alkon, Russell Kirby

**Affiliations:** 1 University of California, Merced, 5200 N. Lake Rd., Merced, CA 95343-5001, USA; 2 University of South Florida, College of Public Health, Tampa, FL, USA; 3 Department of Anthropology, University of South Florida, Tampa, FL, USA; 4 University of California San Francisco, School of Nursing, San Francisco, CA, USA

**Keywords:** Healthy eating, Healthy child care, COVID-19, Mixed-methods research

## Abstract

**Objective:**

The COVID-19 pandemic changed early care and education (ECE) mealtimes. Feeding practices that support children’s emerging autonomy may support children’s healthy eating, but it is unknown whether and how COVID-19 changed feeding practices. This paper describes caregiver feeding practices in ECE centres in Florida during COVID-19.

**Design:**

A mixed-methods design was used to understand mealtime feeding practices. Survey and interview questions were developed based on the Trust Model. More than 7000 surveys were sent to ECE centres. Analysis included descriptive statistics for survey data and thematic analysis for interview data.

**Setting:**

This statewide study included teachers in all licensed and license-exempt ECE centres.

**Participants:**

Four hundred and thirty-one teachers completed a survey, and twenty-nine participated in follow-up interviews.

**Results:**

Surveys showed most teachers engaged in autonomy-supportive behaviours, such as letting children eat until they were finished (90 %). The most common controlling behaviour was praising children for cleaning their plates (70 %). The most common responses about changes to mealtimes were keeping physical distance and serving healthy food. Interview themes were *Autonomy Support, Controlling Feeding Practices, Interactions are the Same, Interactions are Different, Physical Distancing* and *Healthy Eating.*

**Conclusions:**

Mealtimes are a central part of the day for young children and teachers in ECE environments. COVID-19 continues to influence ECE routines as behaviour change remains the primary method of reducing the risk of COVID-19 in the absence of a vaccine for young children. Understanding teachers’ practices and perspectives is important for reducing the risk of COVID-19 and supporting children’s autonomy and healthy eating.

The majority of young children aged 2 to 5 years in the USA attend an early care and education (ECE) programme^(1)^, where teachers play an important role in supporting their development of healthy eating^(2)^. The COVID-19 pandemic brought many changes to ECE routines, including mealtimes, drop-off routines, screening for illness and mask wearing. In Florida, the state child care licensing agency did not provide specific guidance for modifications during mealtimes to reduce the risk of spreading COVID-19^(3)^, although this information was available on the Centers for Disease Control and Prevention (CDC) website for child care programmes^(4)^. The state child care licensing agency did provide new regulations on reducing group sizes and adult-to-child ratios in Spring 2020, but by Fall 2020, these modifications had been lifted^(5,6)^. As the COVID-19 pandemic circumstances became the ‘new normal’, ECE programme directors had to consider mealtime routines and account for both the new risk of COVID-19 and supporting children’s healthy eating.

## Child feeding practices

‘Child feeding practices’ refers to how adults feed children^(7,8)^. The general understanding of child feeding practices in ECE settings is based on food parenting practices, which is based on parenting taxonomies such as Diana Baumrind’s *authoritarian, authoritative* and *permissive/indulgent* scheme^(9)^. Feeding practices that support children’s emerging autonomy are thought to support children’s development of competent eating^(10–12)^. A few studies have looked at child feeding practices in terms of teacher engagement and interactions with the children. One cross-sectional study found that teacher behaviours increased average number of tasted fruits and vegetables and lower number of tasted foods high in fat/sugar when teachers assessed whether children were full before removing their plate, discussed healthy foods during the mealtime and ate the same foods together with the children^(12)^. Similarly, an observational study of twenty-four Dutch ECE centres found that children ate more fruit when teachers talked to them about preparing the meal; children consumed fewer sweets when teachers let them help with meal preparation; and children consumed more vegetables when teachers generally encouraged eating^(13)^. Another observational study found that staff sitting with children and eating with them was positively associated with children eating more vegetables and fewer overall calories^(14)^. All of these studies support the idea that positive adult engagement with children around food and mealtimes has the potential to support children’s healthy and competent eating.

## The trust model

One conceptualisation of supportive child feeding practices is the Trust Model. The Trust Model was first described in the 1980s by Ellyn Satter, a dietician and social worker who worked with children and families with feeding problems/eating disorders. An early publication about the feeding relationship included some content about division of responsibility^(15)^. In later work, she described ‘eating competence’ as a skill to be developed, along with the numerous other competencies young children develop as they grow up (e.g. communication and motor skills)^(16)^. Eneli coined the term ‘The Trust Model’ in a 2008 article that outlined the key elements of the model^(17)^, in which children and adults have separate responsibilities, with context describing the natural growth patterns of any individual child. These ideas re-framed disordered eating into a different lens. What was viewed as a temporary problem to be fixed became a skill to be developed and nurtured on an ongoing basis.

**Fig. 1:**
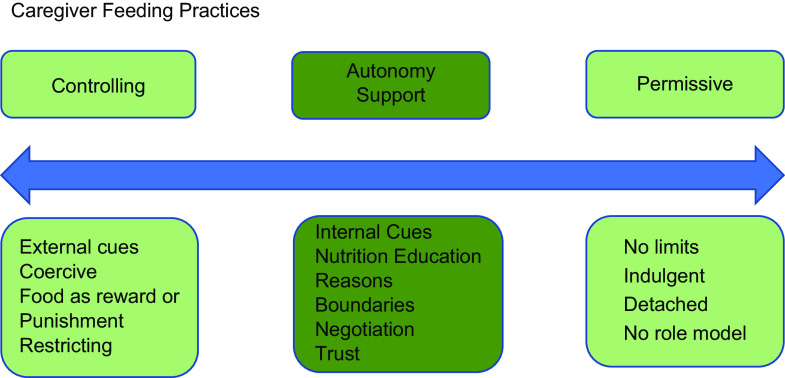
Continuum of child feeding practices *Source*: Baumrind^(51)^.

The Trust Model links to Baumrind’s parenting taxonomy in that more controlling food parenting practices fall within the authoritarian parenting category, autonomy-supportive food practices align with authoritative parenting, and indulgent or disengaged parenting can also be indulgent or disengaged food parenting (Fig. 1). The Trust Model aligns with an authoritative food parenting style, in which the parent is a leader and a figure the child can trust but is neither overly intrusive/controlling nor indulgent/permissive^(18)^. Although the Trust Model was originally designed for the home setting and as a treatment for already disordered eating, the current curriculum is intended to be used from birth^(19)^ and focuses on all caregiver/child feeding interactions^(20)^, supporting the idea that positive feeding behaviours and attitudes are applicable to all children at every stage.

In this study we applied the Trust Model to understand child feeding practices in early education settings during COVID-19. The Trust Model focuses on both adult and child responsibilities, empowering children to decide what and how much they eat with reasonable adult guidance^(17)^. Other models of food parenting practices tend to focus on adult behaviours^(21)^, rather than focusing on the development of an ongoing relationship. However, at school, children are developing relationships with their teachers, who are tasked with supporting their development in many domains, including developing the skill of competent eating. Additionally, within the Trust Model, children are responsible for speaking up or otherwise signalling to their teacher that they are hungry. Finally, children are expected to tolerate hunger for short periods of time because they can trust that food will be available at the usual time and place^(16)^. All of these features of the Trust Model make it suitable for ECE environments, in which children are independent of their parents and/or siblings and learning to communicate on their own, they are learning and developing rapidly across domains, and mealtimes are typically on a predictable schedule. Other studies have also used the Trust Model in ECE settings^(22–24)^.

The purpose of this study was to examine mealtime social interactions among teachers and children in ECE centres in Florida during the COVID-19 pandemic. This paper addresses the following research questions: (1) How did ECE teachers in Florida describe the caregiver-child feeding dynamic during COVID-19? and (2) What changes (if any) did ECE teachers in Florida describe in the caregiver-child feeding dynamic due to COVID-19? The operational definition of teachers for this paper are the ‘caregivers’ in ECE centres.

## Methods

### Study design, participants and setting

A mixed-methods concurrent design was used to describe and understand mealtime practices in ECE centres in Florida during COVID-19. Participants in this paper include only classroom teachers.

Participants were recruited with assistance from the Florida Department of Children and Families. Only director emails were publicly available, and so the survey link was distributed to directors first. Directors were asked to forward the email to one classroom teacher that fit the criteria.

Participants were included if: (1) they were a classroom teacher in an ECE centre in Florida; (2) worked in a licensed or license-exempt ECE centre in Florida; (3) they were responsible for lunchtime in a classroom for children aged 2–5 years; and (4) the director of their ECE centre forwarded the survey link to them.

### Instrument development

#### Survey

A theory-based survey and interview questions were developed based on the Trust Model and existing literature on child feeding practices. The survey was developed both to reflect framing questions in terms of the Trust Model^(17)^ and to incorporate the unique context of COVID-19 (e.g. including questions about food insecurity). The survey was developed first in English and then translated into Spanish by three research professionals who are fluent in Spanish. Two additional research assistants with high proficiency in Spanish took the online survey and provided detailed feedback regarding language, grammar and meaning. Their suggested changes were documented and incorporated into the Spanish-language version of the survey and consent form.

Face validity was established by first requesting feedback from three national experts on healthy eating in ECE. Changes included adjustments such as splitting complex questions into separate questions. Content validity was established by sharing the survey with [JFM] dissertation committee and seminar doctoral students. Changes included clarifying language and adding a question about directors’ decision-making process. Finally, the survey was pilot-tested with four local ECE directors and teachers in [JFM] home county. Directors and teachers were recruited via [JFM] community Facebook page. Participants provided feedback via Facebook Messenger. Their feedback was used to make adjustments to clarify terminology (e.g. ‘school’ and ‘teacher’) and adding one open-ended fill-in question at the end. Finally, two students piloted the Spanish version of the survey and provided two grammatical changes and one change to clarify word usage involving the acronym ‘ECE’. Additionally, the students provided feedback about taking the survey in Qualtrics, such as whether text boxes worked correctly. After incorporating all feedback, the cross-sectional survey was distributed to more than 7000 directors of ECE centres via email in Florida on 10 August 2020 and remained open until midnight on 11 October 2020.

Teachers were asked thirteen questions about their mealtime social interactions with the children. The questions were designed with a six-point Likert scale (Always, Very Often, Often, Sometimes, Rarely and Never). Questions asked how often teachers engaged in mealtime activities that had been defined as either controlling or supportive of children’s autonomy based on a review of the literature.

Teachers were also asked whether COVID-19 had changed their mealtime interactions: ‘Has COVID-19 changed the way you interact with the children during a typical lunchtime?’ The question included eight closed-ended responses, to which participants could select ‘yes’, ‘no’ or ‘other.’ Participants could select more than one response.

#### Interview

A semi-structured, open-ended interview guide, based on Trust Model^(17)^ constructs, was used to interview twenty-nine teachers. Participants were asked to describe mealtime interactions in response to all interview questions, which are listed in Table 1. Specific concepts measured by these questions are based on the child feeding practices literature: *Social Interactions: Controlling* and *Social Interactions: Autonomy Support.* The interview also captured responses related to *Changes due to COVID-19.*

**Table 1:** Interview questions for teachers

Questions
1.a. Tell me about the mealtimes at your school, specifically thinking about lunchtimes. What is it like right now? 1.b. How have mealtimes changed at your school since COVID-19?
2.a. Tell me about your responsibilities as a teacher during the lunchtime. What do you feel responsible for during the lunchtime? 2.b. Do you think that your responsibilities as a teacher during lunchtime have changed since COVID-19? If so, how?
3.a. Tell me about what the children are doing during lunchtimes at your school. What are they responsible for during the meal? 3.b. Do you think that the children’s responsibilities during lunchtime have changed since COVID-19? If so, how?
4.a. How are the children doing in general right now? 4.b. How are the children doing with the social changes due to COVID?
5.a. How are the teachers doing in general right now? 5.b. How are the teachers doing with the social changes due to COVID?

### Data collection procedure

#### Survey

The survey was distributed to more than 7000 ECE directors in Florida. Because teacher email addresses are not public, the survey was distributed to directors and directors were asked to forward the survey to a classroom teacher involved in lunchtime. It was initially sent on 10 August 2020. Subsequent reminders were sent out on 15 September, 23 September, 28 September and 2 October 2020. The survey was closed on 11 October at midnight. Directors were asked to forward the survey to a classroom teacher involved in lunchtime. To minimise the burden of participation in research, directors were asked to select a teacher whom they thought could answer questions about mealtimes. Surveys were distributed via Qualtrics.

#### Interview

At the end of the survey, teachers were invited to participate in a follow-up interview. To have a diverse sample of ECE teachers and make them as representative as possible, we used purposive sampling with multiple steps. The first ten interviewees were selected by contacting all who responded. After the 10th interview, participants were purposefully selected to add unique counties in order to gain as much variation as possible in terms of regions of Florida represented. Sampling was stopped when the data reached saturation. All interviews were completed from 16 August 2020 to October 16, 2020 via phone or Zoom. Participants who completed the interview were compensated with a $25 e-giftcard to Amazon. The Institutional Review Board at the University of South Florida reviewed and approved this study protocol and designated this study as exempt. Participants provided informed consent for the survey via an online form. The consent document with research descriptions including potential risks and benefits of the study was attached in the first page of the survey, and only individuals who agreed to participate were able to proceed with the survey. At the beginning of each interview, a consent script was read and recorded and participants provided verbal consent to participate, and their consent was recorded.

## Data analysis

### Survey

Survey data were analysed using descriptive statistics and frequencies in SPSS version 27.0.

### Interview

For all twenty-nine interviews, the interviewer was the lead author. Interviews were audio-recorded and transcribed *verbatim* before uploading to MAX QDA for thematic analysis. The initial set of codes included *a priori* codes based on the Trust Model^(17)^ and Social Cognitive Theory^(25)^ and used a deductive process to identify concepts. *Social Interactions* and *Changes due to COVID* were *a priori* codes. Inductively, sub-codes including *Controlling* and *Autonomy Support* were defined and refined as these concepts emerged from the data. A second coder, who was also trained extensively in qualitative data analysis, double-coded six transcripts. The lead author and the second coder achieved inter-coder reliability with a *κ* greater than 0·80.

### Data integration

Quantitative and qualitative data were integrated across the key concepts: *Controlling Feeding Practices, Autonomy Supportive Feeding Practices* and *Changes in Caregiver-Child Feeding Practices* (Appendix 1). Interview responses that aligned with each concept were assessed for whether they were consistent with the survey results in each corresponding concept category. Results are presented within these three guiding concepts.

## Results

### Participants

Surveys were completed by 431 teachers (411 in English and 20 in Spanish), representing forty out of sixty-seven counties in Florida (60 %). The twenty-nine interview respondents were from nineteen out of sixty-seven counties (28 %).

Participant demographics were similar to the Florida statewide ECE workforce (Table 2). The majority (96 %) were female, which is consistent with general demographics of the ECE workforce both at the state and national levels^(26)^. Among survey respondents, 24 % reported they were of Hispanic origin, 62 % White and 25 % Black. Additionally, most teachers who responded to the survey were full time, and their hours and employment status had not changed due to COVID-19 (Table 3).

**Table 2:** Teacher gender and race/ethnicity demographics

Gender	Teachers *n* 384	Florida ECE workforce*
Female	96·4	97·4
Male	2·3	2·6
Other	1·3	na
Hispanic ethnicity *n* 382		
Yes	23·8	26·7
No	76·2	69·9
Race *n* 389		
White	62·0	43·2
Black or African American	24·9	26·7
Asian	1·5	0·5
American Indian or Alaska Native	0·5	0·0
Native Hawaiian or Pacific Islander	0·8	0·2
Other (please specify)	10·3	1·6

* The workforce study did not have a gender category ‘other’, and it did not allow respondents to select both ‘White’ and ‘Hispanic.’

**Table 3:** Teacher employment demographics

Employment status	Teachers *n* 385
Full time (30 h or more/week)	88·8
Part time (less than 30 h/week)	11·2
Hours changed	*n* 384
My hours are about the same	71·1
My hours have decreased due to COVID-19	19·5
My hours have increased due to COVID-19	9·4
Employment status changed	*n* 381
My employment status has not changed	88·7
My employment status has changed from full time to part time due to COVID-19	8·4
My employment status has changed from part time to full time due to COVID-19	0·3

### Mealtime feeding practices: autonomy support

#### Survey

Table 4 shows the frequency with which teachers reported several common mealtime interactions. Interactions were either controlling or supportive of children’s autonomy based on the theoretical framework (Fig. 1). In terms of supporting children’s autonomy, nearly all teachers responded allowing children to eat until they were finished, 75 % or more talked about food and non-food topics at the table, and about half or more asked children about their hunger and fullness and used adult and peer role modelling to encourage healthy eating. The only item on which the majority did not respond with autonomy support was about allowing a child not to eat.

**Table 4:** Frequencies of mealtime interactions, autonomy support

Autonomy support	Always,very often and often	Sometimes, rarely and never
	%	
I let children eat until they are finished^(8)^.	90·2	9·9
I talk with the children about food^(10,12,46,32)^.	83·6	16·4
I talk with the children about non-food topics^(14)^.	74·6	25·4
I ask children if they feel hungry^(10,12,17)^.	61·3	38·8
I ask children if they feel full^(10,12,17)^.	58·0	42·0
I encourage children to try a new food by trying it together with them^(47,25,46)^.	53·3	46·7
I encourage children to try a new food by pointing out other children eating the food^(47,48)^.	48·3	51·7
If a child is not hungry, I let them sit through the entire meal without eating^(17,45,35)^.	18·2	81·9

#### Interviews

During interviews, teachers also described mealtime interactions across a continuum. Several concepts from survey questions were consistent with interviews and were described in more detail during the interviews. For example, teachers described children as being free to eat until they were full, engaging with the children about food and non-food topics, and using adult and peer modelling to support children’s healthy eating.

One teacher described an example of supporting children’s autonomy in terms of allowing them to eat until full: ‘So you know, at the dinner table, no matter what age group, including babies, if I sense that they need more or act like they’re still hungry, they can get more to eat’ (Teacher 021).

Other teachers described having conversations about food and non-food topics: ‘they’re still close enough that they can have conversations, we talk about who has fruit in their lunchbox, and “oh [CHILD’S NAME] and another friend both have strawberries”’ (Teacher 014).

Finally, several teachers described examples of adult and peer modelling, which is one way of supporting children’s autonomy by providing a role model to follow rather than simply telling children what to do. One example of peer modelling: ‘and if they see them [other children] eating salad, they’ll eat salad, you know’ (Teacher 016). Similarly, another teacher described how children may try something new if they see another child try it:

‘I would kind of really like the lunchbox kids to eat my food [which is provided by the school] and get used to what’s going to happen in the cafeteria, and also to try something they might be reluctant to try, or watch somebody else eating and go “oh, maybe broccoli with ranch dip is not that bad”’ (Teacher 008)

### Mealtime feeding practices: controlling

#### Survey

The most common controlling behaviour was praising children for cleaning their plates (70 %), followed by requiring children to try one bite of each food item (59 %). Less than half of respondents reported typically engaging in the remaining controlling behaviours, although 40 % reported encouraging children to eat more out of concern they were not getting enough to eat at home (Table 5).

**Table 5:** Frequencies of mealtime interactions controlling

Controlling	Always, very often and often	Sometimes, rarely and never
	%	
I praise children for cleaning their plates^(35,42)^.	70·0	30·0
I require children to try one bite of each food^(47)^.	59·4	40·5
I encourage children to eat more food when I worry they are not getting enough at home^(49)^.	39·6	60·4
I stop children from eating too much of any one food so there will be enough for everyone^(43)^.	6·5	93·5
I encourage children to eat quickly so we have time to transition to the next activity^(11,50)^.	4·9	94·9

#### Interviews

For controlling feeding practices, interviews provided explanation and context for survey questions that received a high percentage of responses. For example, according to the survey, praising children for cleaning their plates and requiring children to try one bite of a new food were common controlling feeding practices. Interviews helped explain why. For example, some interview participants explained that they encouraged children to eat (and not sit through a meal without eating, per the survey) so that the children would be full and not wake up hungry after a nap: ‘You don’t have to eat it, but at least eat your yogurt and maybe a little bit of your chicken or whatever. Because I want them to have something in their stomach [so they won’t] wake up after nap and be starving to death’ (Teacher 013).

Along similar lines, another teacher described how she encouraged children to eat during a mealtime so that they would consume a variety of foods: ‘So the children of course are required to eat. They choose what they eat… as long as they have two different coloured foods on their plate’ (Teacher 007). Finally, some teachers described encouraging children to take ‘no thank you bites’ which means taking one bite of a food and then saying ‘no thank you’ rather than rejecting a food without trying it: ‘We don’t force them to eat, we encourage them to take “no thank you” bites’ (Teacher 012). A key characteristic of these three quotes is that the teachers do not describe their behaviour as controlling, because there is a rationale behind what they are doing.

Finally, an important issue around the lunches sent from home that was not captured on the survey was the order in which foods were presented to the children. Several teachers expressed concern that if children had access to all of the food at the same time, they would eat the unhealthy foods first and not the main meal. For example: ‘You’re not eating those chocolate chip cookies until you eat the meat and cheese and crackers out of your [NAME BRAND LUNCH]’ (Teacher 013). Another explains that she has some children who are ‘picky’ and need encouragement:

I have some children that are very picky eaters. So I have to make sure that they’re eating food because some of them won’t even touch their food, so I’ll encourage them to try even take a bite or two, and then with some encouragement they’ll try but without me encouraging them to try it, they’ll just throw it in the garbage and not eat anything (Teacher 020).

Another teacher described how important it is to know the children in order to make decisions about plating their lunches brought from home in order to ensure that they eat something other than a sweet:

With my heavy eaters that I know, they will eat all their food, because that’s, you know, that’s how they are they typically eat all their food every day, it’s something that I know they’ve eaten before, I’ll go ahead and plate all of it. And they’ll pick and choose and eat it. You know, by the end of lunch, it’s all gone. Regardless. For those who tend to be more picky eaters, or tend to have a sweet tooth, I will hold back whatever the dessert is for lunch (Teacher 024).

These responses illustrate how in some instances, the teachers have to learn the eating behaviours of each child and develop appropriate routines and strategies to influence what the children are eating.

### Mealtime feeding practices: changes

#### Survey

Teachers responded to one question about how COVID-19 had changed their mealtime interactions. Interestingly, the most frequent response was that interactions were basically the same. The next most common response was keeping a distance from the children, which makes sense given that new COVID-19 precautions recommended keeping distance between individuals. Finally, a focus on healthy eating could be related to beliefs that healthy nutrition would support healthy immune system function and reduce susceptibility to COVID-19 infection, especially at the beginning of the pandemic when there was not a vaccine^(27)^. Responses about parent concerns were not frequently selected, which could reflect the lower amount of contact between parents and teachers relative to pre-COVID-19 times (Table 6).

**Table 6:** Changes in mealtime interactions

Has COVID-19 changed the way you interact with the children during a typical lunchtime? (please select all that apply) (*n* 432)*	*n*	%
No, my interactions with the children during the meal are basically the same.	185	42·8
Yes, now I do not get as close to the children to avoid sharing germs.	140	32·4
Yes, now I encourage the children to eat more healthy foods so that we will all stay healthy.	118	27·3
Yes, now I spend time helping children with their masks before and after eating.	83	19·2
Yes, we used to eat together ‘family style’, but now I do not eat together with the children.	69	16·0
Yes, parents have more concerns about their children eating healthy foods at school.	37	8·6
Yes, now I encourage the children to clean their plates more often so that we do not waste food.	27	6·3
Yes, parents have more concerns about their children eating enough food at school.	25	5·8
Yes, now I bring additional food for children I know are not getting enough to eat at home (e.g. crackers to add to a child’s lunch).	24	5·6
Other, please describe:	21	4·9

* Multiple selections allowed.

#### Interviews

Interviews were consistent with survey results in that many described their mealtime interactions with the children as being generally similar compared with pre-COVID-19. Several participants described small changes that were not difficult to adjust to. Participants also talked about changes in terms of keeping a distance from the children and putting more space between them. They also described encouraging healthy eating in the midst of the COVID-19 changes. Although the percentage who responded on the survey that they help children with masks seems low, it is consistent with approximately the percentage of centres that required masking for children^(28)^.

#### Interactions are the same

Several teachers described changes in mealtimes as small and not a big disruption. For example: ‘More cleaning, more washing hands, and more distance… before we were close to each other, and [the children] pass the spoons and they help the teacher and that changed. But the other things no they are still the same’ (Teacher 003). Similarly, one teacher described spacing the children out as the only real change to their mealtime: ‘before this they used to bring their own lunch so that has stayed the same. What has changed is the fact that they don’t sit that many children per table’ (Teacher 006). Another sums up that the changes are small and do-able:

But you know, surprisingly, like, it’s really not that hard. You know, it’s just a different routine. And, and that’s it, everybody just has to get accustomed to a little bit different routine. If it’s going to make everybody safer, you know… I think is a good thing…it’s not that hard, we can do this (Teacher 011).

#### Interactions are different

Other teachers described some of the changes in mealtime routines due to COVID-19: ‘Before the kids were more hands-on as far as even the cutting up process. They would get the spoons, they would get the forks, they would set up the cups, they would set up the napkins, but now, you know they can’t touch any of that stuff’ (Teacher 004). Another teacher described how the children used to line up together for their food, but now they have to sit and not touch anything while the teachers bring the food:

Now as opposed to lining up for their food, they sit down. And we bring them the food, to the table. Before they could walk around, it was more sociable, you know they would talk and share with each other, and get this and get that. But now they sit down, I serve them at the table, and everyone has to have their hands in their laps until they get their food (Teacher 001).

Another described how her ability to role model for the children had changed due to COVID-19:

We do not, we remain kind of separated from them. Like for me, I sit at my desk and eat, where normally I would sit with the children at the table and model the table manners and behaviour and things like that, and interact more with them. But because of the restrictions of the social distancing and the masks and everything, we’re encouraged not to (Teacher 022).

#### Physical distancing

Many teachers described the physical distancing during mealtimes that is recommended during COVID-19. ‘Now we have all the kids separate at tables, we have two kids per table, [at] each corner’ (Teacher 003). Similarly: ‘the students for the most part they, um, don’t wear a mask for lunch, they are spaced out 6 feet apart, and so that has [decreased] the amount of students we have’ (Teacher 004).

Another teacher described how she continued to eat together with the children but moved her chair away from the group to comply with COVID-19 guidelines:

Yes, yes, I have my chair so I sit far away I try to stay that you know the distance [6 feet] and yeah we are still eating together I don’t use the table because I have to sit in the corner, and I just hold my food. Before I [used to] sit with them at the table and eat with them but now, I just eat in my chair and move back my chair and use it there (Teacher 003).

Another teacher described how she makes an effort to stay away from the children’s food as an explanation for why they don’t sit together anymore: ‘We’re trying to avoid touching anything, um we don’t, I don’t sit down with them like I used to… I try not to get anywhere near their food, um, unless I need to’ (Teacher 002). These comments about keeping distance reflect both compliance with CDC guidelines and appropriate concern about spreading COVID-19 via food.

#### Healthy eating

Some teachers commenting on using different strategies to encourage healthy eating at the table. One described a change in the mealtime interactions in that she no longer served as a role model for healthy eating:

That I don’t get to um [pause] that I don’t get to model that we’re going to eat healthy, I don’t get to interact as much about what they’re eating because I can’t really – I can talk to them when they’re eating, but I try not to. We’re trying not to hover and we don’t really want them talking too much when they’re eating (Teacher 008).

Another described how she changed from role modelling to providing verbal encouragement for healthy eating due to COVID-19:

‘So for this particular group they are not really eating at all. Our main task is to try and encourage them to eat the food, and so we have been telling them, you know, “eat your food so you’ll get big and strong, big and strong” and so we’ve been doing that more and more to encourage them to eat because before the pandemic the teachers we used to eat together with the child, to help encourage the students to eat’ (Teacher 004).

## Discussion

COVID-19 brought unexpected changes to ECE settings across daily routines. Mealtimes are of particular concern because the virus spreads via droplets, which come from mouths and noses, which cannot be covered while eating. Masks and physical distance presented challenges for teachers during mealtimes.

### Role modelling

Role modelling has been shown to be important for children’s eating behaviours, both at home^(29,30)^ and at school^(31)^. In our study, some teachers indicated that their overall mealtime routines had changed in ways that precluded role modelling altogether (such as changing from eating with the children to supervising them). However, other teachers described modifications that both protected against COVID-19 (e.g. moving their chair back and away from the children) while simultaneously adjusting mealtime behaviours to include support for healthy eating practices, such as encouraging children’s healthy eating verbally rather than role modelling. Adjustments such as these, in which teachers still expected children to be responsible for what and how much they eat, reflect some teachers’ knowledge of healthy eating practices and consistency with the division of responsibility that is central to the Trust Model^(17)^. The full implications of some children losing their adult role model have yet to be seen, but this is a loss of one element in the early learning environment that supports children’s healthy eating.

### Child involvement

Although surveys indicated that most teachers perceived routines to be largely the same during COVID-19 as they were before, interviews suggested some significant changes, including changes in children’s involvement in the mealtimes. Involving children in the mealtime, such as talking with children during meal preparation and letting children help get the meal ready, has been shown to encourage healthier eating^(32)^. Future work could look more closely at programmes in which children’s involvement in the mealtime diminished due to COVID-19 and assess whether and how this may have influenced children’s healthy eating.

### Controlling feeding practices

Some teachers described behaviours that could be considered controlling feeding practices, which is one possible behavioural response to food scarcity. However, they were often rationalised within a set of rules, which would be predictable for the children. The literature is not clear on whether controlling feeding practices in ECE is necessarily as ‘bad’ for children as they are at home. Some studies find that authoritative feeding practices, like authoritative parenting, is the preferred style that will promote children’s healthy eating^(33,34)^. However, other studies indicate that the ECE setting may be different in ways that are important to what feeding style is most supportive. One study found that permissive feeding behaviours by ECE caregivers was associated with children eating more vegetables^(11)^. Another paper articulates that ECE teachers have a unique role and are not part of the children’s families, and therefore interactions and expectations are different^(35)^. Teachers at school are in a different role from parents at home. Additionally, children at school are part of a same-age peer group (typically, in the USA), not a family system that may involve siblings of various ages^(35)^. Also, the ECE food environment is much more rigid than home food environments. Meals and snacks are served on a schedule, and children do not have independent access to food while at school (unlike at home). The structure of the ECE environment is predictable, and therefore, the effects of food rules on children’s eating behaviours must account for the differences in their environments.

### Emergent issue

A final emergent issue is that several teachers talked about putting out foods brought from home in specific ways to control which foods some children ate first. Additionally, some teachers indicated that their school used a mix of mealtime styles, in that some children brought food from home while others received meals at school. Given that some studies have found the nutritional quality of parent-send lunches to be less than ideal^(36–38)^, and that changing to a parent-send food model could be a reasonable decision for centres during COVID-19, the topic of how teachers serve parent-send meals is another area of further exploration. Future studies should also include the ‘mixed’ style of meal service given that peer modelling is one important aspect of how children learn to eat.

### Policy implications

The policy landscape in the USA is complex in that while federal programmes provide meals for ECE settings with children from low-income households, the majority of ECE settings are regulated by state standards, and there is variation across the states in how they address mealtime practices (both before and during COVID-19). While federal programmes such as Head Start and the federal Child and Adult Care Food Program (CACFP) encourage evidence-based healthy eating practices, including adult role modelling and children serving themselves^(39,40)^, many states’ regulations do not. While some states do have language indicating that CACFP rules should be followed, (a) there is little oversight^(41)^ and (b) ECE staff may interpret such regulations inconsistently^(42)^. While CACFP acted quickly to try and make sure children received food when their schools were closed during COVID^(43)^, millions of children attend ECE settings that do not participate in CACFP. Therefore, our findings indicate that closer alignment between state regulations and national standards would provide more consistency in following healthy mealtime practices.

### Limitations and strengths

This study has several limitations. First, the data were collected at only one point in time and relied on respondents’ ability to assess changes retrospectively. The interviewer is not fluent in Spanish and so interviews were conducted only in English, potentially creating bias in the group of interview respondents. Future work should include interviewers who can conduct the interview in Spanish and other common languages (such as Haitian-Creole in Florida).

The COVID-19 pandemic created some unique limitations in data collection: for example, participation could have been biased towards individuals with time and interest in participating. Along similar lines, this study could only include participants from centres that were open and does not represent centres that were closed. The number of teacher surveys was relatively low due to the difficulty in distributing the survey to teachers via directors. Also, directors could have selected teachers who shared similar beliefs about child feeding practices, although it is likely that feeding practices would be similar across rooms in a centre. Although the authors conducted face and content validity for the survey questions, more rigorous validity testing, such as criterion and construct validity^(44)^ and confirmatory factor analysis^(45)^ would be a beneficial future direction.

Despite these limitations, this study provides a novel contribution to the field of caregiver feeding practices in that it examines experiences during an infectious disease pandemic. Lessons learned could be applied to similar large-scale system disruptions, such as natural disasters or other major social disruptions.

## Conclusion

Mealtimes are a central part of the day for young children and teachers in ECE environments. COVID-19 will continue to influence ECE routines as behaviour change remains the primary method of reducing the risk of COVID-19 among young children. Future research should include direct observation of mealtimes. Findings from this study show that there is a range of controlling and autonomy-supportive feeding behaviours happening during COVID-19, which means that it is possible to support children’s autonomy during this pandemic. Classroom teachers could benefit from the professional support of having an additional adult in the room during mealtimes given additional pandemic precautions and increased tasks. Continuing to support children’s emerging autonomy during COVID-19 will be essential for children developing healthy eating habits and supporting their overall health.

## Supplementary material

For supplementary material accompanying this paper visit https://doi.org/10.1017/S1368980022002452. click here to view supplementary material
