# Supplementary material for: Mixed-method assessment of caregiver feeding practices in early care and education centres during COVID-19
Source: Public Health Nutr. 2022 Nov 8:1–11. doi: 10.1017/S1368980022002452 (PMC9744446; doi:10.1017/S1368980022002452)
Supplement: Supplementary file 1 [file S1368980022002452sup001.docx]

# Appendix A: Survey for Teachers (English)

**Today’s Date _________________ School ID ____________________**

| **SECTION ONE: GENERAL INFORMATION** |
| --- |

*This set of questions will ask about some general information.*

(1) What is your county? _____________

(2) What is your 5-digit zip code? ______________

(3) Do you regularly participate in lunchtimes at your school?

Yes _____

No _____ **[if No, skip to end]**

| **SECTION TWO: TYPICAL LUNCHTIME SETTING** |
| --- |

*This set of questions will ask about your typical lunchtime setting* ***currently*** *for children age 3-5 years, eating normal “table food.” “Currently” means your typical daily practice during the past week.*

(4) Where do the children in your class usually eat lunch **currently**?

- Indoor Classroom ______
- Indoor Cafeteria ________
- Outdoors _______
- Other ____________________________________________________________

(5) During lunchtime with your class **currently,** about how many children eat together at the same time? 1-10 _____ 11-20 _____ 21-30 _____ 31-40 _____ 40+ _____

(6) About how many tables are in the eating area **currently**?

1 ___ 2 ___ 3 ___ 4 ___ 5+ ___

(7) About how many children sit at each table **currently**?

1-5 ____ 6-10 ____ 11-15 ___ 15+ ___

(8) About how many teachers participate during lunch with your class **currently**?

1 ___ 2___ 3 ___ 4 ___ 5+ ___

| **SECTION THREE: TYPICAL LUNCHTIME ROUTINES** |
| --- |

*This set of questions will ask about your typical lunchtime routines* ***currently*** *for children age 3-5 years, eating normal “table food.”*

(9) **Currently,** how much time do children have to eat lunch? (Please think about when the first child in a group begins and the last child in the same group ends.) **[drop down menu]**

(10) Do children eat lunch at the same time each day? Yes _____ No _____

(11) Do children eat lunch in the same location each day? Yes _____ No _____

(12) Which best describes the lunchtime routine in your classroom **currently** (please select only one):

- Children serve themselves most foods, and children decide how much to take _____
- Children serve themselves most foods, and adults decide how much children may take __
- Adults serve most foods, and children decide how much to take _____
- Adults serve most foods, and adults decide how much to give to children _____
- Food arrives at the school already portioned on each child’s plate _____
- Children bring food from home _____

| **SECTION FOUR: ANY CHANGES IN LUNCHTIMES** |
| --- |

*This set of questions will ask whether and how your typical lunchtime routines* ***have changed*** *since February 2020 (before COVID-19) for children age 3-5 years, eating normal “table food.”*

(13) Have your lunchtime routines changed due to COVID-19? (please select all that apply)

(a) Yes, we changed **where children sit** (e.g., putting more space between children) ____

(b) Yes, we changed **where children eat** (e.g., changed from cafeteria to classroom) ___

(c) Yes, we changed the **source of our meals** (e.g., changed from kitchen-onsite to parent bring) _____

(d) Yes, we changed the **types of foods** that are served (e.g., serving more healthy foods, serving less expensive food) _____

(e) Yes, we are doing **more cleaning** activities during mealtimes (e.g., increased handwashing and/or sanitizing surfaces) _____

(f) No, mealtime routines are basically the same _____

(g) Other, please describe: __________________________________________________

________________________________________________________________________

| **SECTION FIVE: TEACHERS’ MEALTIME RESPONSIBILITIES** |
| --- |

*This set of questions will ask about your responsibilities as a teacher during a typical lunchtime* ***currently.***

(14) Do you sit with the children during lunch?

- - Yes, during the entire mealtime _____
  - Yes, during part of the mealtime _____
  - No _____

(15) Do you eat the same foods as the children during lunch?

Yes _____

No, I eat different foods _____

No, I do not eat while the children are eating _____

(16) Are all lunch items served at the same time? Yes _____ No _____

**[If no]** (16a) Which items are served first? _________________

**[If no]** (16b) Which items are served later? _________________

(17) For meals **currently,** do parents ever ask you to feed their child in a specific way (e.g.,

please make sure my child finishes his/her lunch?)

Yes, often _____ Sometimes _____ Rarely _____ Never _____

(18) For meals **currently,** do parents ever ask you to feed their child certain foods (e.g., please make sure my child eats his/her vegetables?)

Yes, often _____ Sometimes _____ Rarely _____ Never _____

(19) Are additional servings of food available during lunch?

Yes, additional servings of food are available for **all items** _____

Yes, additional servings of food are available for **some items** _____

No, additional servings are not available _____

(20) Is milk served during lunch? Yes _____ No _____

**[If Yes]** (20a) Is additional milk available during lunch? Yes _____ No _____

(21) Is water served during lunch? Yes _____ No _____

(22) Do you have additional responsibilities during lunchtime? (e.g., paperwork, planning, preparing nap mats, etc.) Yes _____ No _____

(23) Do you wear a mask (i.e., cloth or disposable face covering that covers your mouth and nose) during any of the following times **before lunch?** (please select all that apply)

When you are getting ready for lunch (e.g., setting the table, preparing plates of food) __

When you are helping the children wash their hands before lunch _____

When you are serving any food to the children _____

When you are helping children get out any food brought from home _____

When you are helping the children remove and store their masks _____

No, you do not wear a mask during any of these times before lunch _____

Other (please describe): ____________________________________________________

(24) Do you wear a mask during any of the following times **after lunch?** (please select all that apply)

When you are helping children clean up the lunch table _____

When you are helping children wash their hands _____

When you are helping children get ready for their nap _____

No, you do not wear a mask during any of these times after lunch _____

Other (please describe): ____________________________________________________

| **SECTION SIX: CHILDREN’S MEALTIME RESPONSIBILITIES** |
| --- |

*This set of questions will ask about the children’s responsibilities during a typical lunchtime* ***currently.***

**(25) How often do children decide:**

|  | Never | Rarely | Sometimes | Often | Very Often | Always |
| --- | --- | --- | --- | --- | --- | --- |
| (a) whether to eat? |  |  |  |  |  |  |
| (b) what to eat? |  |  |  |  |  |  |
| (c) how much to eat? |  |  |  |  |  |  |
| (d) what **not** to eat? |  |  |  |  |  |  |

**(26) How often do children decide:**

|  | Never | Rarely | Sometimes | Often | Very Often | Always |
| --- | --- | --- | --- | --- | --- | --- |
| (a) whether to drink? |  |  |  |  |  |  |
| (b) what to drink? |  |  |  |  |  |  |
| (c) how much to drink? |  |  |  |  |  |  |
| (d) what **not** to drink? |  |  |  |  |  |  |

**(27) How often do children tell you:**

|  | Never | Rarely | Sometimes | Often | Very Often | Always |
| --- | --- | --- | --- | --- | --- | --- |
| (a) when they feel hungry? |  |  |  |  |  |  |
| (b) when they feel full? |  |  |  |  |  |  |

(28) Do the children wear a mask during any of the following times **before lunch?** (please select all that apply)

When they are getting ready for lunch (e.g., setting the table, getting lunch boxes) ____

When they are washing their hands _____

When they receive their food _____

No, the children do not wear masks during any of these times before lunch _____

Other (please describe): ____________________________________________________

(29) Do the children wear a mask during any of the following times **after lunch?** (please select all that apply)

When they are cleaning up (e.g., throwing away trash, putting away lunchbox) _____

When they are washing their hands _____

When they are getting ready for nap _____

No, the children do not wear masks during any of these times after lunch _____

Other (please describe): ____________________________________________________

| **SECTION SEVEN: MEALTIME SOCIAL INTERACTIONS** |
| --- |

*This set of questions will ask about your social interactions with the children a typical lunchtime* ***currently.***

**(30) How often do you do the following with the children during lunchtime?**

|  | Never | Rarely | Sometimes | Often | Very Often | Always |
| --- | --- | --- | --- | --- | --- | --- |
| (a) I praise children for cleaning their plates. |  |  |  |  |  |  |
| (b) I require children to try one bite of each food. |  |  |  |  |  |  |
| (c) I ask children if they feel hungry. |  |  |  |  |  |  |
| (d) I ask children if they feel full. |  |  |  |  |  |  |
| (e) I stop children from eating too much of any one food so there will be enough for everyone. |  |  |  |  |  |  |
| (f) I encourage children to eat more food when I worry they are not getting enough at home. |  |  |  |  |  |  |
| (g) I let children eat until they are finished. |  |  |  |  |  |  |
| (h) I encourage children to try a new food by trying it together with them. |  |  |  |  |  |  |
| (i) I encourage children to try a new food by pointing out other children eating the food. |  |  |  |  |  |  |
| (j) I encourage children to eat quickly so we have time to transition to the next activity. |  |  |  |  |  |  |
| (k) I talk with the children about food. |  |  |  |  |  |  |
| (l) I talk with the children about non-food topics. |  |  |  |  |  |  |
| (m) If a child is not hungry, I let them sit through the entire meal without eating. |  |  |  |  |  |  |

| **SECTION EIGHT: CHANGES IN MEALTIME SOCIAL INTERACTIONS** |
| --- |

*This set of questions will ask about how your social interactions with the children during lunchtime* ***may have changed*** *due to COVID-19.*

(31) Has COVID-19 changed the way you interact with the children during a typical lunchtime? (please select all that apply)

(a) Yes, now I spend time helping children with their masks before and after eating. ____

(b) Yes, now I encourage the children to clean their plates more often so that we do not waste food. _____

(c) Yes, now I encourage the children to eat more healthy foods so that we will all

stay healthy. _____

(d) Yes, now I bring additional food for children I know are not getting enough to eat at home (e.g., crackers to add to a child’s lunch). _____

(e) Yes, now I do not get as close to the children to avoid sharing germs. ____

(f) Yes, we used to eat together “family style,” but now I do not eat together with the children. _____

(g) Yes, parents have more concerns about their children **eating healthy** foods at school.___

(h) Yes, parents have more concerns about their children **eating enough** food at school. _

(i) No, my interactions with the children during the meal are basically the same. _____

(j) Other, please describe: __________________________________________________

(32) Is there anything else you want to share about how COVID-19 has influenced mealtimes at your school? ___________________________________________________________________

____________________________________________________________________________________________________________________________________________________________

| **SECTION NINE: DEMOGRAPHICS** |
| --- |

*In order to make sure that we hear from a variety of people, this section will ask some questions about your demographic information:*

(D1) Do you currently work Full Time or Part Time?

Full Time (30 hours or more per week) _____

Part Time (less than 30 hours per week) _____

(D2a) Have your hours changed due to COVID-19?

Yes, my hours have increased _____

Yes, my hours have decreased _____

No _____

(D2b) Has your employment status changed due to COVID-19?:

Yes, my employment status has changed from Full time to Part time _____

Yes, my employment status has changed from Part time to Full time _____

No _____

(D3) In what year were you born? (please use 4 digits, e.g., 1985, 1970, etc.) ____________

(D4) What is your gender? Female _____ Male _____ Other _____

(D5) Are you Hispanic or Latinx? Yes _____ No _____

(D6) What is your race? (These categories are from the US Census. Please select all that apply.)

- - American Indian or Alaska Native _____
  - Asian _____
  - Native Hawaiian or Pacific Islander
  - Black or African American _____
  - Caucasian/White _____
  - Other (please specify): ______________________________________________

(D7) How many years of experience do you have working in Early Care and Education? ______

(D8) What is your educational background? (please select all that apply)

- - Florida ECE Staff Credential _____
  - Florida ECE Director’s Credential _____
  - National Child Development Associate (CDA) _____
  - Tier 1-5 Certification __________
  - High school diploma or GED _____
  - Some college education _____
  - Associate’s Degree _____
  - Bachelor’s Degree _____
  - Master’s degree or higher _____
  - Other: ____________________________________________________________

**Thank you for filling out this survey!** I know that ECE programs can be very different and each individual’s experience is unique. To help me understand your point of view, would you be willing to participate in a follow-up phone interview? I can provide a $25 e-gift card to Amazon for your participation. Yes _____ No ______

**If Yes, please provide your email and/or phone number and I will get in touch to schedule an interview: __________________________________________________________**
